# Role of Lipids on Entry and Exit of Bluetongue Virus, a Complex Non-Enveloped Virus

**DOI:** 10.3390/v2051218

**Published:** 2010-05-18

**Authors:** Bishnupriya Bhattacharya, Polly Roy

**Affiliations:** London School of Hygiene & Tropical Medicine, Keppel Street, London WC1E 7HT, UK; E-Mail: priya.bhattacharya@lshtm.ac.uk

**Keywords:** BTV, non enveloped, lipid rafts, entry, exit

## Abstract

Non-enveloped viruses such as members of *Picornaviridae* and *Reovirida*e are assembled in the cytoplasm and are generally released by cell lysis. However, recent evidence suggests that some non-enveloped viruses exit from infected cells without lysis, indicating that these viruses may also utilize alternate means for egress. Moreover, it appears that complex, non-enveloped viruses such as bluetongue virus (BTV) and rotavirus interact with lipids during their entry process as well as with lipid rafts during the trafficking of newly synthesized progeny viruses. This review will discuss the role of lipids in the entry, maturation and release of non-enveloped viruses, focusing mainly on BTV.

## 1. Introduction

The replication cycle of viruses involves entry into host cells, synthesis of viral genes and proteins, assembly of progeny virus particles and their subsequent egress or release. Along with the plasma membrane, viruses also have to interact with the endosomal and vesicular membranes during their replication in host cells. All cellular membranes are composed of lipids and proteins that are usually arranged in various micro domains. During infection of cells by enveloped viruses, the lipids present in both viral and cellular membranes mediate fusion and fission reactions to facilitate virus entry and egress. Since non-enveloped viruses do not have a lipid envelope, it is generally believed that their entry mechanism does not involve membrane fusion activity and that these viruses are mainly released by cell lysis. Usually, non-enveloped viruses enter the cells by penetrating the membrane barrier, either via the endocytic pathway using clathrin-coated vesicles or caveolae, or by the formation of a pore at the cell surface [1–4]. Recent data obtained from biochemical and structural studies indicate that the overall mechanisms of both entry (*Reoviridae*) and release of certain non-enveloped viruses (e.g., members of the *Picornaviridae* and *Reoviridae*) are analogous to that of enveloped viruses, and that the capsid proteins can function in these activities in a similar manner to the membrane viral proteins. As an example, in this report we will discuss mainly the interaction of Bluetongue virus (BTV) proteins that form a separate genus in the non-enveloped *Reoviridae* family with membrane lipids both during the virus entry and the exit.

## 2. BTV morphology and its entry process into the host cells

Orbiviruses (some 140 members) which form a genus within the *Reoviridae* family, infect animals, plants and insects, and are transmitted by arthropod vectors. An important factor in the distribution of BTV worldwide is the availability of suitable vectors, usually biting midges (gnats) of the *Culicoides* genus. BTV infects wild and domestic ruminants, particularly sheep often with high morbidity and mortality. Bluetongue disease (BT) was first observed in domestic animals in Africa in the late 18th century. Currently, BTV (24 serotypes) is endemic in many parts of the world, particularly in tropical and subtropical countries. As a result of its economic significance, BTV has been the subject of extensive molecular, genetic and structural studies and is now the most well characterized orbivirus.

The virion particle is composed of seven discrete proteins (VP1-VP7) that are organized into two concentric shells (capsids); an outer shell and an inner shell or ‘core’ and a genome of 10 dsRNA segments [5]. The outer capsid, consisting of two major structural proteins, VP2 and VP5, forms a continuous layer that covers the inner core that is composed of two major proteins (VP3 and VP7) and three minor enzymatic proteins (VP1, VP4, and VP6). Shortly after infection, BTV is uncoated (removal of VP2 and VP5) to release the transcriptionally active core particles into the cytoplasm. The structure of the core is well characterized both by cryo-electron microscopy analysis and X-ray crystallography [6–9]. Hence, much is known about the core proteins and their structure-function relationship. In contrast, the atomic structure of neither the complete virion particle nor the outer capsid proteins is available to date. Until recently, the only structural information available for the outer capsid proteins and the whole virion were generated from two different cryo-EM studies, one at very low resolution and the other at a relatively higher resolution [10,11]. These data gave limited understanding of how the two proteins may function during virus entry into cells. However, very recent high resolution cryo-EM studies have thrown some new light on the structure of the two outer capsid proteins and how they may take part in virus entry.

In mammalian cells, BTV entry proceeds via virus attachment to the cell, followed by endocytosis and release of a transcriptionally active core particle into the cytoplasm. Of the two outer capsid proteins, the larger VP2 (110 kDa) is the most variable and is the serotype determinant of the virus. Indeed, VP2 is responsible for eliciting neutralizing antibodies and possesses haemagglutination activity of BTV. Further, VP2, which oligomerizes as a trimer, binds to receptor(s) on the plasma membrane of mammalian host cells and is also responsible for virus internalization [12].

Some limited information regarding BTV entry was initially generated based on ultra-structural electron microscopy studies, which indicated that BTV entry utilizes clathrin coated vesicles [13]. Therefore, it was necessary to undertake a more detailed analysis at a molecular level that combined both confocal microscopy and biochemical studies [14,15]. Initially, the adoption of the clathrin-mediated pathway was investigated by using a siRNA specifically designed to target the protein μ2, which is a subunit of the AP2 complex [16]. The main function of this protein complex is to recruit clathrin proteins from the cytoplasm and to bring them to the plasma membrane to form the clathrin pits [17,18]. The effect of μ2 depletion on BTV entry was independently monitored by immunofluorescence and biochemical studies. Data obtained from both investigations clearly indicated a direct correlation between the restriction of the clathrin pathway and BTV entry. Confocal microscopy revealed that depletion of transferrin uptake in HeLa cells by μ2 siRNA also resulted in a similar reduction of BTV uptake. The retention of BTV particles on the plasma membrane of cells lacking the endosomal vesicles proved that BTV internalization is dependent on clathrin. Further evidence regarding the role of clathrin in BTV entry was provided by measuring the virus replication of the cells that were infected with BTV after siRNA transfection. The results from these experiments demonstrated reduction of BTV replication was directly associated with the absence of the clathrin-pathway. A different approach involved the use of chlorpromazine (CPZ) to block the formation of the clathrin vesicles. This antibiotic directly interferes with the generation of the clathrin pits and hence its effect is very specific. Different sets of experiments have revealed that increasing amounts of CPZ during BTV infection resulted in inhibition of virus replication. Confocal microscopy analysis of the infected cells supported further that the reduction of BTV infectivity was not due to chemical toxicity. The data acquired by μ2 siRNA and CPZ treatments clearly demonstrated that BTV enters the cells using the clathrin-mediated pathway [15]. This particular system of endocytosis usually proceeds with the delivery of the cargo contained in the clathrin vesicles to the early and the late endosomes. While influenza virus uses the acidic environment of the late endosomes to become fusion competent, for the non-enveloped adenoviruses, the low pH of the early endosomes triggers conformational changes of a viral protein required for cell permeabilization ability [19]. For both viruses, the final aim of the mechanism is to release the virion into the cytoplasm. The relevance of the acidic pH in BTV entry was initially suggested by Hyatt *et al*. [20], who showed that the presence of ammonium chloride (NH_4_Cl) inhibited BTV replication. The pH-dependent character of BTV entry was further examined more recently by monitoring the effect of bafilomycin A1 in BTV infectivity [15]. This chemical has been classified as a vacuolar type H^+^-ATPase inhibitor, and is responsible for the specific inhibition of endosomal acidification, blocking endosomal traffic during endocytosis. From our studies, it was clear that the effect of bafilomycin A1 in BTV replication was strictly correlated with the reduction of virus infection in a dose-dependent manner. In parallel, confocal microscopy supported the hypothesis that the endosomes are the perfect location for BTV activation leading to virus uncoating. To further prove that BTV is internalized by the endosomal vesicles and to understand the internalization route followed by the virus, specific endosomal markers were used to investigate the presence of the virus in the early and late endosomes. The results obtained from various studies demonstrated that in the early stages of the infection BTV is present in the early endosomes for at least 30 minutes before further progression of the virion to the late endosomes [15]. Since the assay monitored the virus by labeling VP5 the second outer capsid protein, it is possible to speculate that, due to the membrane binding activity of the protein, it remains attached to the membrane of the early endosomes while the core is released into the cytoplasm. This pathway of virus entry probably relies on specific conformational changes that occur in the BTV outer capsid in response to the changing environment of the virion as it enters the endocytic pathway.

**Figure 1.**
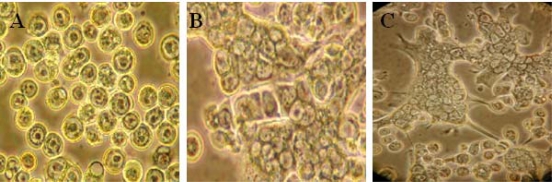
Fusogenic activity of VP5. *Sf9* cells were infected for 48 hours and were then exposed to pH 5.0. Pictures were taken before **(A)** and after 4 hr **(B)** or 7 hr **(C)** pH shift.

The 60 kDa protein, VP5, shares certain secondary structural features with the fusion proteins of enveloped viruses, indicating that it may be responsible for membrane penetration activity [21]. Two distinct biochemical studies support the membrane penetration activity of VP5; peptides representing the two amino terminal amphipathic helices cause leakiness of the membrane and the full length VP5 protein, when localized to the plasma membrane, triggered strong syncytia and formation of multinuclear cells (Figure 1) upon low pH treatment [14,21]. Thus, VP5 has the capacity to destabilize membranes and it does so after it has been activated by low pH conditions that are similar to the endosomal environment encountered during cell entry. In addition, VP5 also forms a trimer in solution when expressed as a recombinant protein, a feature shared by other fusion proteins. It is likely that upon receptor binding and entry, VP2 trimers undergo rearrangement or degradation allowing the exposure of VP5 trimers which subsequently changes to the functional conformation due to low pH. VP5 lacks the autocatalytic cleavage and N-terminal myristoyl group present in the entry proteins of reoviruses and rotaviruses and does not require proteolytic activation in contrast to some other viral fusion proteins [22]. How exactly VP2 and VP5 may interact to the lipid membrane during cell entry became clearer further from the structural data recently obtained from the whole virion particle.

## 3. BTV outer capsid structure

**Figure 2.**
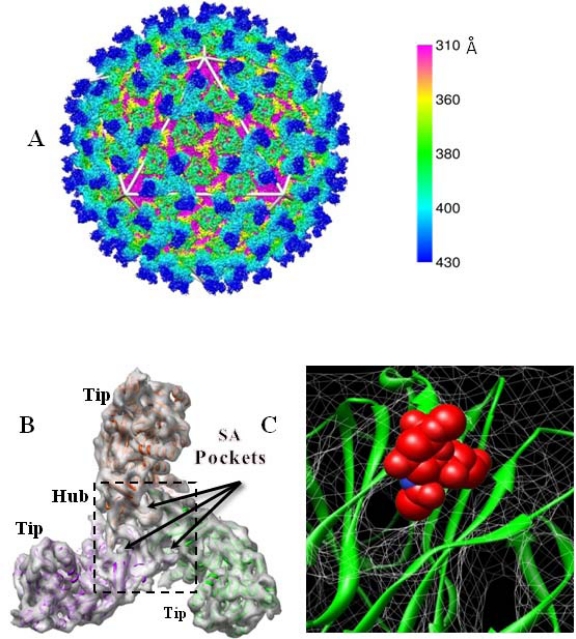
Cryo-EM analysis of BTV particle at 7 Å. **(A)** Cryo-EM structure of complete BTV particle at 7 Å resolution, color coded by radial position: VP2 (blue) and VP5 (green) of outer capsid; VP7 (pink) of core. **(B)** Top view of VP2 triskelion formed by three VP2 monomers, each consisting of a tip domain and a part of the hub domain as indicated. A putative SA-binding pocket is located at the hub domain. **(C)** Fitting of SA into the pocket. Courtesy X. Zhang *et al.* [24]

**Figure 3.**
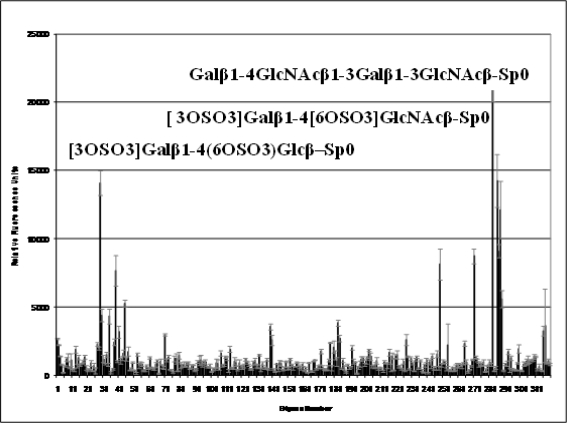
Glycan array analysis of VP2 binding. Version 3.0 of the printed array of the Consortium for Functional Glycomics was probed with VP2 (200 μg/ml). *Error bars* represent the mean ± standard error of four replicates. Glycans yielding significant binding are shown with their numbers and names.

The BTV outer capsid has an icosahedral configuration with a diameter of ∼880 Å [11]. A series of three-dimensional structural studies, using cryo-EM (initially at 40 Å and subsequently at 25 Å resolution) have been undertaken. The reconstructed images revealed the overall organization of both the VP2 and VP5 trimers in the outer capsid, which consisted of a total of 60 triskelion spike-like structures formed by VP2 trimers and 120 globular VP5 trimers. Together, they covered most of the underlying core layer although both VP2 and VP5 trimers attach to the underlying core surface layer independently of each other [10,11,23]. However, a recent 7 Å resolution cryo-EM structure of BTV virions (Figure 2A) has identified the secondary structural elements and the topological arrangement of these two proteins in more details [24]. Each of the three monomers of VP2, which assembles as a triskelion structure, has two distinct domains: a tip domain and a distant lower region, three of which form a hub domain at the base. The top of the tip domain of each monomer is rich in β sheets and projects upward from the surface of the virion and its location implies that this region is responsible for the first encounter of the virus with the host cell membrane. The hub domain which is formed by all three monomers, on the other hand, has a distinct β-barrel fold (Figure 2B) that is similar to the sialic acid (SA) binding domain of the outermost region (VP8) of the rotavirus VP4 spike [25]. Further, when the ribbon model of the rotavirus SA-binding domain was docked into the VP2 density map it fitted perfectly into this hub region. The putative sialic acid binding region was further confirmed by fitting a SA into a pocket in this site (Figure 2C). Thus, even though there is no sequence homology between the rotavirus VP8 and the BTV VP2, these structural findings suggest that VP2 binds SA and this was further confirmed using biochemical approaches. Our recent data confirmed that VP2 binds type 2 oligosaccharide structures (Galβ1-4GlcNAcβ1-3) in glycan arrays (Figure 3). In BTV VP2 the SA binding domain is about 40 Å inward from the outer end of the tip domain, a distance well within the range of lengths of chains of sugars on glycoproteins. Hence after attachment of the tip domain to the host cell surface, the SA binding of VP2 might further stabilize the interaction, thus facilitating infection of host cells. This SA binding of VP2 has been recently confirmed biochemically and in confocal studies by infecting cells with BTV in the presence of Wheat Germ Agglutinin (WGA), a SA binding protein. This study revealed that not only was there a decrease in the total titer but also that the cells were comparably less infected. However, as the infection was not completely abolished in the presence of WGA, other receptors might also be influencing BTV entry. Since it is known that BTV particles agglutinate erythrocytes of ruminants [26] and VP2 alone is responsible for this activity [21], it can be postulated that SA binding may promote adsorption of the virus onto the surface of erythrocytes, thereby increasing the probability of ingestion by the blood-feeding *Culicoides* vector.

**Figure 4.**
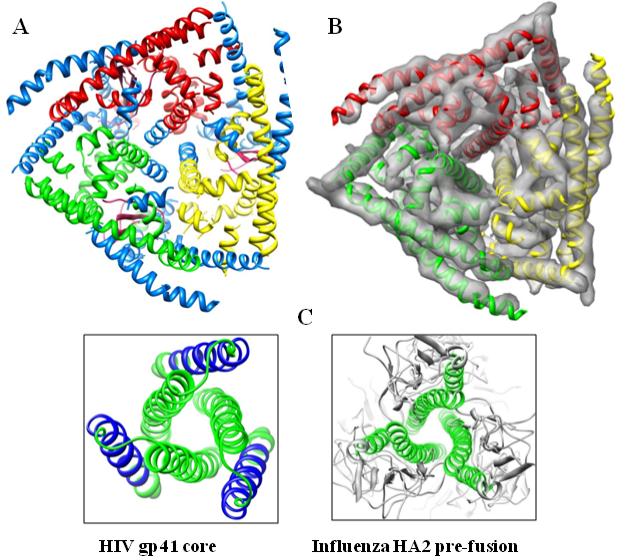
Cryo-EM analysis of VP5 trimer at 7Å resolution reveals it as a helical globular complex. **(A)** The top view of a VP5 trimer showing the amphipathic helices (blue) and three non-amphipathic helices (red, green, and yellow). **(B)** Six-fold average density map of the same with an embedded ribbon model. The density map reveals secondary structures demonstrating that VP5 contains many helices but only one β-sheet. **(C)** Ribbon diagrams of the central coiled-coil helix bundle and a triplet of peripheral helices in fusion proteins gp41 of HIV and HA2 of influenza. Blue ribbons represent amphipathic regions of α-helices; green ribbons represent non amphipathic regions of α-helices. Courtesy X. Zhang *et al.* [24].

The structural data revealed that the globular VP5 is very rich in helices and possesses only one β sheet (Figure 4A). The N-terminal amphipathic α helical region is positioned on the exposed surface of VP5 and there are four additional amphipathic α-helical regions that are also located on the exterior surface of the protein [24]. In particular, three copies of one of the four amphipathic helices present on the top surface are well positioned to expose their hydrophobic undersides to endosomal membrane (Figure 4B). In addition, the other three amphipathic helices are similarly located on the exposed surface. Interestingly, similar peripheral triplet of α helices is present in HIV trimeric fusion protein [27]. Another trimer of α helices in the VP5 trimer that forms a coiled-coil helix bundle and runs up the center of the trimer is analogous to what is commonly observed in the fusion proteins of the enveloped HIV (Figure 4C) [28], influenza virus (Figure 4D) [29], herpesvirus [30] and VSV [31], as well as in the penetration protein of the non-enveloped rotavirus [32]. The gaps in the VP2 triskelion are filled by the three VP5 trimers.

Density maps have also revealed that the VP2 trimers interact (only via hub domains, not tip domains) with VP5 trimer very weakly, and similarly the interactions between VP5 and VP7 trimers are also very weak. These weak interactions would permit conformational changes of VP5 during the penetration process and during shedding of the outer coat. In comparison, both the VP2 tip and hub domains connect to their underlying VP7 trimers by stronger forces.

## 4. BTV replication and assembly

Removal of the outer capsid proteins activates the virion transcriptase [33,34] in the virus cores and results in the synthesis of capped mRNAs of each genome segment that are then extruded from the cores into the cytoplasm where they serve as templates for translation of viral proteins [33]. Thus, the core structure consisting of all the necessary enzymes (provided by the three minor proteins) is responsible for synthesizing mRNAs [33–39].

EM analysis of thin sections of BTV infected cells have revealed the presence of small numbers of intracellular virus particles and large numbers of juxtanuclear fibrillar networks referred to as virus inclusion bodies (VIBs). These structures are composed predominantly of non-structural phosphoprotein NS2 that recruits all the components of subviral particles (subcore), *i.e.,* the viral ssRNAs, transcriptase complex (TC) proteins (VP1, VP4 and VP6) as well as VP3 that encapsidates the TC and the viral genome [40–47]. The polymerase protein VP1 in the newly formed subcores is then believed to synthesize the negative strand RNAs on the ssRNA templates to produce genomic dsRNA [39]. The subcore in the VIBs serves as a scaffold for the addition of VP7 trimers, thereby giving rise to more rigid and stable cores [47–49]. Similar inclusion bodies are also present in rotavirus and reovirus infected cells [50–52]. However, the outer capsid proteins are not recruited by NS2 and are not assembled within the VIBs [45,47]. Both the proteins appear to be assembled onto the core in a different location than the VIBs.

## 5. Interaction of outer capsid proteins of BTV with lipid rafts

**Figure 5.**
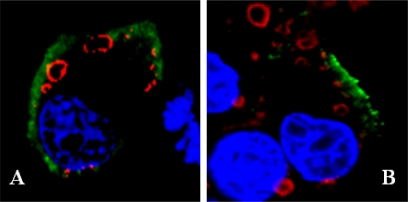
Confocal microscopy shows VP5 at the plasma membrane of BTV infected cells. BSR cells were infected with BTV and analyzed at 12 **(A)** and 16 **(B)** hours post infection. VP5 was stained with anti-guinea pig FITC (green), BTV NS2 with anti-rabbit TRITC (red) and nuclear DNA with Hoechst stain (blue).

In infected cells at later times post-infections, both VP2 [53] and VP5 (Figure 5) [54] can be seen at the plasma membrane by confocal microscopy. Recent biochemical data also suggests that VP2 [53] and VP5 [54] interact with the raft domains in the cellular membrane. Lipid rafts are dynamic microdomains in cellular membranes and are formed by the segregation of membrane lipids like sphingolipids and cholesterol in a glycerophospholipid-rich environment [55,56]. Many viruses and other pathogens hijack lipid rafts to facilitate their successful infection [57–59]. Usually, proteins interact with rafts either via their transmembrane domains [60] or through covalent lipid modifications [61]. For enveloped viruses, generally fusion proteins associate with lipid rafts in cellular membranes. Thus, it is not surprising that VP5, which acts like a fusion protein, has this property. However, VP5 is not myristoylated although it contains a conserved glycine residue at a position suitable for modification [3].

The association of VP5 with raft domains is quite convincing, since depletion of cellular cholesterol with methyl beta cyclodextrin not only altered the association of VP5 with raft domains, but also decreased the relative BTV viral titer, indicating the importance of raft domains in BTV replication [54]. In addition, VP5 possesses a WHXL motif, a highly conserved domain that is also present in a SNARE regulatory protein Synaptotagmin I (Syt1) [62]. This domain has been shown to be responsible for docking of the cellular protein in the plasma membrane. Interestingly, when the WHXL of VP5 was substituted with a series of alanines, the association of VP5 with lipid rafts was severely perturbed and it was no longer localized at the plasma membrane but was visible as patches in the cytoplasm [54]. In comparison to VP5, VP2 does not contain any obvious lipid raft binding domains, thus it is still not clear the exact mechanism of interaction between VP2 and lipid raft domains. However, VP2 interacts with vimentin [63] which itself interacts with both microtubules and lipid rafts. Thus it may be that the association of vimentin acts as a bridge in the association of VP2 with lipid rafts.

Similar interactions have been observed for rotavirus and lipid rafts during the later stages of virus replication. Although rotavirus specifically infects highly polarized intestinal cells *in vivo* [64], the majority of research in rotavirus assembly has been undertaken in non-differentiated MA 104 cells. Subsequent to the assembly of the double layered particles (DLPs) in inclusion bodies, termed as “viroplasm”, DLPs bud out from the viroplasm, (a process that is facilitated by a viral non-structural protein, NSP4) and acquire the outercapsid proteins VP4 and VP7. Both VP7 and NSP4 are inserted in the ER membrane co-translationally and recent data suggests that the assembly of VP7 layer onto the DLPs takes place in the ER [65]. NSP4 has also been localized with plasma membrane raft protein caveolae in some cells [66].

In contrast to VP7, the other outer capsid protein of Rotavirus, VP4, is cytosolic. However, it associates with lipid rafts in both MA 104 cells and Caco 2 cells but this association is sensitive to cholesterol extraction by methyl beta cyclodextrin only in one cell line but not the other [67]. Since VP4 does not exhibit an obvious transmembrane domain nor does it have a GPI (glycosylphosphatidylinositol) anchor and neither is it glycosylated, it has been speculated that its interaction with lipid rafts is most likely via an alternative method. VP4 possesses two potential lipid raft binding domains in its sequence, a caveolin binding consensus motif and a flotillin binding motif. Both caveolin and flotillin are lipid raft proteins. Although the caveolin binding domain of VP4 is a suitable candidate for raft interaction, the absence of caveolin in Caco-2 cells [68] raises questions regarding rotavirus-lipid raft interactions in polarized intestinal cells. In addition, there is no experimental data to date to support the activity of the flotillin binding motif present in VP4 although it has been proposed that it might be responsible for its interaction with rafts [69]. However, it has been proposed that since the amino terminal end of the VP4 has a galectin-like fold, this might interact with raft domains in cells thus behaving in analogous manner to cellular galectin [69].

In infected Caco-2 cells, VP4 associates with cellular lipid raft domains early in infection [70]. Since other rotaviral structural proteins also associates with rafts, it was proposed that the final step of rotavirus assembly is an extrareticular event. This was further confirmed by the interaction of rotaviruses (rhesus and murine strains) with lipid rafts during infection *in vitro* and *in vivo* [71]. Studies analyzing the effect of inhibitors of cholesterol biosynthesis also suggested that absence of cellular cholesterol results in poor incorporation of viral proteins into virions [72].

## 6. Release of BTV from infected cells; role of NS3 protein

**Figure 6.**
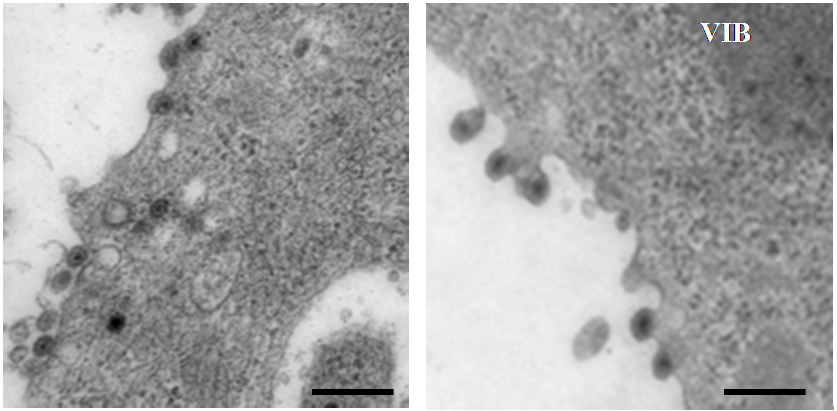
Ultrastructural analysis of BTV infected cells. BSR cells were infected, fixed at 12 hours post infection, and processed for cell sectioning. Note: virus particles are underlying the plasma membrane (left panel) or budding from the cell membrane (right panel) of infected cells. Presence of virus inclusion bodies (VIBs) is indicated. Bar = 100 nm.

**Figure 7.**
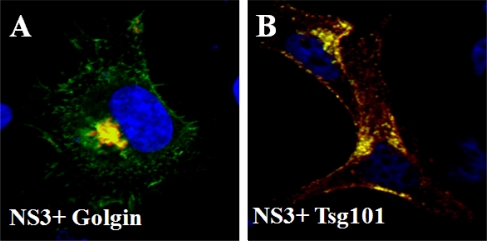
Localization of NS3 with cellular markers. Co-localization (yellow) of NS3 with Golgi marker Golgin **(A)** and Tsg101 **(B)** in transfected cells. The proteins are indicated in each panel.

BTV infects and replicates successfully both in mammalian and insect cells. Although BTV is released from infected mammalian cells mainly by cell lysis, it also egresses either by budding or protrusion and the nature of release depends on the particular cell type (Figure 6). In infected cells, virus particles were observed along intermediate filaments by EM studies [73] and this phenomenon has been further confirmed more recently [63]. Interestingly, along with virion particles, the smallest non-structural protein NS3, the only glycosylated protein of BTV, has also been observed with cellular membranes together with VP2 and VP5, the two outer capsid proteins [54,74,75]. NS3 has also been localized to the ER, Golgi complex (Figure 7A) and to the actin [76,77]. Proteins that are synthesized in the ER are transported to the Golgi where they are sorted in the trans-Golgi network (TGN) for delivery to early/sorting endosomes. They may be then transported to late endosomes/ multi vesicular bodies (MVBs) that are important in the segregation of proteins and lipids destined for (a) lysosomal degradation (b) recycling to the Golgi, and (c) plasma membrane exocytosis. Among the cellular proteins NS3 directly interacts with Tsg101 (Figure 7B), a component of MVBs (the transport vesicles of late endosome), and annexin II complex involved in exocytosis, respectively [75,78–80]. By using recombinant proteins it was shown that Tsg101 interaction is mediated by a conserved PSAP motif in NS3 which appears to play a role in virus release. Depletion of Tsg101 with siRNA also inhibited the release of BTV [80].

More recently using a reverse genetic system we confirmed that viruses containing mutations of the Tsg101 binding motif had altered patterns of virus egress and left nascent particles tethered to the cellular membrane, indicating that Tsg101 binding of NS3 is directly involved in pinching off the newly assembled viruses from the plasma membrane [81]. Further, cells infected with mutant viruses incapable of NS3-VP2 interaction not only perturb the budding process of the virion particles but also arrest all the particles in the cytosol [81].

In subcellular fractionation experiments, not only does NS3 co-fractionate with raft domains in virus infected cells but cholesterol-dependent raft integrity is also essential for NS3 segregation with rafts [54]. Since cholesterol is synthesized in the ER and Triton X-100 fractionation methods have identified cholesterol/sphingolipid-enriched structures in the trans-Golgi network and lysosomes [82,83], it can be postulated that NS3 uses these microdomains for transit from the ER to membranes. Lipid rafts are thought to arise from the Golgi apparatus, where sphingolipids are synthesized and to which they recruit proteins destined for apical trafficking in epithelial cells, as proposed by Simons and Ikonen in “the raft hypothesis” [84]. Although, NS3 does not have any raft interacting domain, it is glycosylated and has two transmembrane domains that might be guiding its interaction with the raft domains. In enveloped viruses, localization of the viral glycoprotein occurs at the site of virus budding. Although, for most enveloped viruses their glycoproteins contain specific signals that result in targeting, or retention, at the budding site, other viral factors also determine the budding of enveloped viruses such as VSV [85,86] and Measles virus [87,88].

Similar to BTV, rotavirus release also depends on cell type. While the virus is released by lysis in MA104 cells, it exits by non-lytic means from the apical surface of differentiated cells [89–91]. Since disruption of lipid raft integrity by cellular cholesterol depletion also decreased virus release from infected cells it was concluded that rotaviruses uses these domains to transport to the cell surface during replication [91].

## 7. Conclusions

Membranes provide a physical barrier both in cell entry and egress of viruses from infected cells. Compared to the enveloped viruses that have a lipid bilayer that enables fusion of virus and cellular membrane, the non-enveloped naked capsids must adopt alternate strategies to penetrate through the cellular membrane lipid bilayer. In BTV this function is achieved by VP2 and VP5, the outer most virus structural proteins

Structural studies of VP2 suggest that following the attachment of the tip domain to the cell surface, secondary binding to sialic acid via the hub domain might cement the interaction and lead to cell ingress [24]. BTV then enters the host cell by a clathrin-dependent endocytic pathway. Once within the cellular endosomes the adjacent globular VP5 trimer layer is exposed to the acidic pH and becomes fusiogenic. It has been proposed that the amphipathic helical regions present on the outer surface of VP5 could swing up to the membrane and subsequently roll to make extensive hydrophobic contact that perforates the membrane to release the transciptionally active cores into the cytoplasm. This unfurling of VP5 could detach it and the rest of the VP2 from the core [24]. The importance of amphipathic helices in insertion of proteins into the lipid membrane bilayer has also been revealed for the μ1 and VP4 proteins of reovirus and rotavirus, respectively. Recently it has been reported that the conformational changes of rotavirus VP4 influence its interaction with the lipid bilayer which in turn might influence subsequent stages of viral core penetration from the endosome into the cytoplasm [92]. The similarity of the location and the sequence of the key domain of the rotavirus fusion protein with a cluster of three hydrophobic loops of Semliki Forest virus has led to the hypothesis that the disruption of the endosomal membrane might involve insertion of the hydrophobic apex of this domain into the membrane bilayer [93].

Traditionally, it has been believed that non-enveloped viruses are released by cell lysis. However, it has been shown that along with BTV, rotavirus, simian virus 40, poliovirus, and parvovirus can be released without lysis of the infected cells [ [92]. This indicates that naked capsid viruses can behave similarly to enveloped viruses and interact with cellular membranes during virus release.

During BTV assembly, recent studies have shown that along with VP2 and VP5, the non-structural protein NS3 also associates with lipid rafts. The fact that extraction of cellular cholesterol decreases viral titer is consistent with the hypothesis that this interaction is important for the assembly of the outer capsid proteins onto the core particles to form the mature virus particle.

The presence of a transient envelope like structure associated with BTV in EM sections of infected cells has led to the hypothesis that BTV may acquire an envelope via NS3 during virus release. It is noteworthy that BTV and related orbiviruses, seadornaviruses and coltiviruses are the only groups of arboviruses that lack a lipid envelope. It is possible that orbiviruses were originally also enveloped but lost their stable lipid membrane at some point during evolution.
